# Assessing gender dysphoria in Turkish adolescents: psychometric validation of the Utrecht Gender Dysphoria Scale–Gender Spectrum

**DOI:** 10.1192/bjo.2025.10846

**Published:** 2025-10-01

**Authors:** Sabide Duygu Uygun, Esra Yurumez, Yağmur Özgür-Karabıyıkoğlu, İrem Kar, Didem Behice Öztop

**Affiliations:** Department of Child and Adolescent Psychiatry, Faculty of Medicine, Ankara University, Ankara, Turkey; Department of Child and Adolescent Psychiatry, Ankara Bilkent City Hospital, Ankara, Turkey; Department of Biostatistics, Faculty of Medicine, Ankara University, Ankara, Turkey

**Keywords:** Gender dysphoria, psychometric properties, Utrecht Gender Dysphoria Scale–Gender Spectrum (UGDS-GS), adolescents, validity and reliability

## Abstract

**Background:**

Gender dysphoria is linked to various psychosocial challenges in adolescence, underscoring the need to identify and support youth experiencing gender-related distress. Although gender identity exists on a spectrum beyond the binary, no validated tool currently exists in Turkey that uses inclusive, gender-neutral language to assess it in adolescents.

**Aims:**

This study aimed to evaluate the psychometric properties of the Turkish adaptation of the Utrecht Gender Dysphoria Scale–Gender Spectrum (UGDS-GS) among clinical- and community-based adolescents.

**Method:**

A total of 240 participants aged 12–23 years were included. The validity of UGDS-GS was assessed through content validity and confirmatory factor analysis. Reliability was measured using Cronbachʼs alpha and test–retest intraclass correlation coefficient (ICC). A sociodemographic data form, UGDS, UGDS-GS, Rosenberg Self-Esteem Scale (RSES) and Youth Self-Report (YSR) were utilised.

**Results:**

Findings demonstrated strong content validity, with a content validity Index of 0.69, and robust construct validity, indicated by a comparative fit index of 0.993 and a root-mean-square error of approximation of 0.071 following the exclusion of three items. UGDS-GS effectively differentiated scores across demographic groups, showing significant variances based on assigned gender and age. The scale also exhibited excellent criterion validity, evidenced by an area under the curve of 0.947 in receiver operating characteristic analysis, with high sensitivity (80%) and specificity (95.9%) at an optimal cut-off value of 42.50. With a Cronbachʼs alpha of 0.935, UGDS-GS demonstrated strong internal consistency and substantial test–receiver operating characteristic retest reliability (ICC 0.884), alongside notable but weak correlations with several RSES subscales and low to moderate correlations with YSR scores.

**Conclusions:**

These results affirm that tUGDS-GS is valuable and reliable in assessing gender dysphoria in Turkish adolescents. Further research is warranted to improve applicability in diverse contexts and populations.

Gender identity is an individualʼs deeply held sense of their gender, which may or may not align with the gender assigned at birth.^1^ In the case of incongruence, it can lead to significant distress requiring clinical intervention.^2^ Because gender has traditionally been understood within the binary framework of biological and social constructs (as male and female), it is often challenging for individuals experiencing gender incongruence to clearly express this through their appearance, behaviour, speech or clothing.^3^ This can further increase distress and make individuals vulnerable to mental health issues.

Diagnostic classification systems define the distress and discomfort experienced by an individual due to the incompatibility between their expressed or experienced gender identity and the gender assigned at birth as ‘gender dysphoriaʼ or ‘gender incongruenceʼ.^4^ Gender identity can exist at any point along a continuum rather than strictly as male or female.^1^ However, diagnostic criteria are primarily shaped around the individualʼs clear desire to belong to the opposite gender in the binary system.^2^ Screening scales were theoretically developed on the binary structure on which these diagnostic systems are based, which presents difficulties and limitations in the clinical assessment of gender dysphoria.^5^

There are a limited number of scales available to measure gender dysphoria in adults and adolescents, including the Bem Gender Role Inventory, the Cross-Gender Questionnaire, the Utrecht Gender Dysphoria Scale (UGDS), the Gender Preoccupation and Stability Questionnaire and the Gender Identity/Gender Dysphoria Questionnaire (GIDQ).^1,6–10^ However, their use does not seem possible for individuals with non-binary gender identities, such as those who identify as ‘transgenderʼ, ‘gender non-conformingʼ or ‘gender queerʼ. Only a few scales, such as GIDQ and the original UGDS, have been demonstrated to be valid and reliable for Turkish adults.^11,12^ There is currently no valid and reliable tool for evaluation of gender dysphoria in children and adolescents in Turkey.

The original UGDS is a validated, 12-item screening measure widely used to assess gender dysphoria in both adults and adolescents.^7,13^ It has two versions, one for female-to-male and another for male-to-female individuals, and is also utilised for follow-up after medical and surgical interventions.^7^ The necessity to switch from one version to another for the same patient post-surgical treatment complicates standardisation. Additionally, the application of these versions is not suitable for non-binary individuals who do not identify strictly as male or female. Therefore, there is a need for a tool that can be used longitudinally without creating such issues and that can be applied independently of the gender assigned at birth.

In response to this gap, McGuire et al adapted the Utrecht Gender Dysphoria Scale–Gender Spectrum (UGDS-GS) from the binary gender versions created by Cohen-Kettenis and Van Goozen.^7,14,15^ UGDS-GS is a unified and inclusive tool that assesses gender dysphoria for individuals of all gender identities across the spectrum, employing gender-neutral language throughout.^14,15^ However, the Turkish adaptation of UGDS-GS has not yet been conducted, and no tool exists in Turkey for evaluation of gender dysphoria within a non-binary framework. This study aims to assess the psychometric properties of the Turkish adaptation of UGDS-GS in a sample of clinical- and community-based adolescents. Additionally, it seeks to investigate clinical features including self-esteem, internalising problems and externalising problems in adolescents who screened positive for gender dysphoria based on UGDS-GS.

## Method

### Procedure

The working group that created the scale authorised validation of UGDS-GS in Turkey. Two child and adolescent psychiatrists, fluent in English (S.D.U. and E.Y.), translated UGDS-GS into Turkish. Subsequently, the scale was back-translated into English without any knowledge of the original text. The authors compared both the translated and back-translated version with the original English version and noted a few discrepancies. Minor corrections were made by consensus.

All procedures of the study were approved by the Ankara University School of Medicine Human Research Ethics Committee (approval no. İ05-347-23), and the study was conducted in accordance with the Helsinki Declaration of 1975 as revised in 2013. Adolescents aged 12–18 years who attended the Department of Child and Adolescent Psychiatry at Ankara University Medical School, and first- and second-year medical faculty students aged 17 to 23 years, were invited to participate in the study. The age range of 12–23 years was chosen to reflect the broad developmental continuum of adolescence and emerging adulthood, which is increasingly recognised in contemporary psychiatric and developmental literature. While traditional definitions often restrict adolescence to ages 10–19 years, recent frameworks – including those endorsed by the World Health Organization and developmental psychologists – acknowledge the extension of key adolescent transitions (e.g. identity formation, emotional regulation, social role experimentation) into the early twenties.^8^ Therefore, participants aged 17–23 years, especially those in their first years of university, were considered developmentally comparable for the purposes of this study. After receiving detailed information about the study, written informed consent was obtained from all participants. For individuals under the age of 18 years, additional parental consent was also obtained in accordance with the requirements of the ethics committee.

Adolescents were administered the sociodemographic data form, the Turkish versions of UGDS and UGDS-GS, the Rosenberg Self-Esteem Scale (RSES) and the Youth Self-Report (YSR).

### Measures

#### Sociodemographic data form

This form was developed by the researchers to collect relevant sociodemographic information about the sample. It included questions regarding the adolescentʼs age, assigned gender and educational status. Furthermore, it gathered data on parental age, educational level and family income.

#### UGDS

The original scale, developed by Cohen-Kettenis and Van Goozen, measures gender dysphoria and comes in two versions: one for male-to-female and one for female-to-male.^7^ Both versions of the scale utilise a 12-item, unidimensional, Likert-type format, where respondents rate each item on a scale from 1 (‘agree completelyʼ) to 5 (‘disagree completelyʼ). A Turkish adaptation of the scale was conducted by Turan et al.^16^ The Cronbach alpha coefficient of UGDS was found to be 0.964 for the trans women group and 0.894 for the trans men group.

#### UGDS-GS

This was adapted by McGuire et al to assess gender dysphoria regardless of biological sex.^14^ It consists of 18 items with a 5-point Likert system, ranging from 1 (‘disagree completelyʼ) to 5 (‘agree completelyʼ). UGDS-GS has two subscales: gender affirmation (items 1, 3, 4 and 5) and dysphoria (items 2, 6–18), with no items reverse coded. The scale is appropriate for longitudinal use from adolescence to adulthood and can be administered at any stage of the social or medical transition process, making it suitable for both research and clinical applications focusing on gender dysphoria for all gender identities and expressions.^15^

### RSES

This scale, developed by Morris Rosenberg, consists of 63 items distributed across 12 subscales including self-esteem, stability of self-concept, trust in others, sensitivity to criticism, depressive affect, daydreaming, psychosomatic symptoms, perception of threat in interpersonal relationships, degree of engagement in discussions, parental involvement, relationship with father and feelings of psychic isolation. The self-esteem subscale comprises ten questions that utilise a four-point Likert-type format, with five of the items being reverse coded.^17^ A high score on the self-esteem subscale indicates low self-esteem. These subscales can be used independently in research, facilitating an in-depth exploration of different facets of self-esteem and interconnected concepts. The scale was later adapted to the Turkish context, maintaining a total of 63 items.^18^ The validity coefficient was determined to be 0.71 and the reliability coefficient 0.75.^19^

### YSR

The YSR serves to standardise the assessment of competence areas and problem behaviours based on self-reports from adolescents.^20^ This scale comprises 17 competence items and 112 problem behaviour items, paralleling the constructs found in the Child Behavior Checklist for Ages 4–18 (CBCL 4–18). Notably, the items are written in the first person to enable accurate self-evaluation by adolescents. The first section of the scale assesses competence through subscales for activities, social functioning and school performance. Activity items evaluate adolescentsʼ participation and success in sports and extracurricular activities, as well as the number of tasks they complete at home and elsewhere. Social functioning items consider club memberships, the number of close friends and the ability to manage tasks independently. School-related items reflect academic success and involvement in school events. The overall competence score is calculated by summing the scores from the activity, social functioning and school subscales. Additionally, there are open-ended questions regarding physical health, school concerns and personal strengths, which are not scored. The second section aligns with CBCL 4–18 and the Teacherʼs Report Form, containing 112 problem items of which 89 are common across all 3 instruments. Problem behaviours are rated based on their occurrence over the past 6 months using a scale of 0 (‘not trueʼ), 1 (‘sometimes or somewhat trueʼ) and 2 (‘very true or often trueʼ). Results yield scores for internalising, externalising and total problems, with internalising scores reflecting social withdrawal, somatic complaints and anxiety/depression, and externalising scores capturing delinquent and aggressive behaviours. Total problems encompass social, thought and attention problems that do not fall under the previous categories. The Turkish adaptation of the scale has reported Cronbachʼs alpha coefficients of 0.80 for internalising issues, 0.81 for externalising issues and 0.89 for total problems, demonstrating satisfactory internal consistency.^21^

### Statistical analyses

Statistical analyses were conducted using IBM SPSS Statistics (version 11.5 for Windows; IBM Corp., Armonk, NY, USA; see https://www.ibm.com/spss), and the validity–reliability metrics were calculated with the ‘caretʼ package in R (version 6.0-94 for Windows; R Core Team, R Foundation for Statistical Computing, Vienna, Austria; see https://cran.r-project.org/package=caret). Descriptive statistics are presented as mean ± standard deviation and median (first quartile – third quartile) for continuous variables, while categorical variables are expressed as frequency (percentage). The validity and reliability of the Turkish UGDS-GS were evaluated, and the corresponding criteria were computed. The construct validity of UGDS-GS was assessed using confirmatory factor analysis (CFA). To evaluate model fit, goodness-of-fit indices were employed, including the comparative fit index (CFI; >0.90, acceptable; >0.95, excellent), Tucker–Lewis Index (TLI; >0.90, acceptable; >0.95, excellent) and the root-mean-square error of approximation (RMSEA; <0.08, acceptable; <0.05, excellent). Known group validity was assessed by examining the scaleʼs ability to reveal expected differences based on specific patient parameters, which were analysed using the Mann–Whitney *U*-test and Kruskal–Wallis variance analysis. Criterion validity was assessed through receiver operating characteristic (ROC) curve analysis, with UGDS serving as the gold standard. Reliability was measured by internal consistency using Cronbachʼs alpha coefficient, and test–retest reliability was assessed through the intraclass correlation coefficient (ICC), along with its 95% confidence interval, calculated from scores obtained from the same participants at two different time points. Internalising problems, externalising problems, self-esteem and related variables based on YSR and RSES in adolescents screened positive and negative for gender dysphoria according to UGDS-GS were compared using the Mann–Whitney *U*-test. A *P*-value of <0.05 was considered statistically significant.

## Results

A total of 240 participants were included in the study, 168 of whom were female (70%), with a mean age of 17.4 years (s.d. = 2.22). Table 1 presents the sociodemographic characteristics of the participants. To assess the content validity of UGDS-GS, a total of 40 experts in child and adolescent psychiatry participated in the evaluation. These experts were recruited by sending email invitations through professional networks in Turkey; although the invitation was distributed to a broader group, 40 experts responded and completed the assessment. Each expert independently evaluated the relevance of every item to the construct of gender dysphoria using a 3-point scale, where 1 is ‘essentialʼ, 2 is ‘useful but not essentialʼ and 3 is ‘not necessaryʼ, in accordance with Lynnʼs method for content validity analysis.^22^ This method emphasises that items rated as ‘essentialʼ by a sufficient proportion of experts are considered to demonstrate adequate content validity. Based on these ratings, the Item-Level Content Validity Index (I-CVI) was calculated for each item, and the Scale-Level Content Validity Index Average (S-CVI/Ave) was determined as 0.69. According to Lynn, a S-CVI/Ave of 0.67 or higher is considered acceptable when the number of experts exceeds 6.^22^ Thus, all items were retained in the item pool. However, the S-CVI/Ave of 0.69, although acceptable, is slightly below the commonly recommended threshold of 0.75, suggesting potential room for refinement. Informal qualitative feedback from several experts indicated that some items pertaining to gender affirmation may have been challenging due to limited familiarity with gender-neutral terminology in the Turkish cultural and clinical context. The lowest I-CVI observed was 0.40, which, while low, did not warrant exclusion, particularly given the conceptual importance of the item and the exploratory nature of the cultural adaptation process.

**Table 1:** Sociodemographic characteristics of participants

Variable	Clinical-based		Community-based		Total sample	
	Mean ± s.d.	Median (Q1–Q3)	Mean ± s.d.	Median (Q1–Q3)	Mean ± s.d.	Median (Q1–Q3)
Age (years)	15.52 ± 1.52	16 (14–17)	19.25 ± 0.79	19 (19–19)	17.4 ± 2.22	18 (16–19)
Gender, % (*n*)						
Female	75% (90)		65% (78)		70% (168)	
Male	24.2% (29)		35% (42)		29.6% (71)	
Intersex	0.8% (1)		0% (0)		0.4% (1)	
Educational status (years)	10.06 ± 1.51	10 (9–11)	13.1 ± 1.66	13 (13–14)	11.59 ± 2.19	12 (10–13)
Maternal age (years)	43.13 ± 5.32	43 (39–47)	46.35 ± 5	46 (43–50)	44.67 ± 5.4	45 (41–49)
Maternal education (years)	10.37 ± 4.04	12 (5–12)	11.78 ± 5.64	16 (5–16)	11.07 ± 4.93	12 (5–16)
Paternal age (years)	46.52 ± 5.81	46 (42–50.5)	49.91 ± 4.4	50 (47–52)	48.2 ± 5.41	48 (45–52)
Paternal education (years)	10.94 ± 4.03	12 (8–14)	13.5 ± 4.21	16 (12–16)	12.19 ± 4.31	12 (8–16)
Family income level	32 706.06 ± 22 388.54	25 000 (20 000–37 250)	41 537.31 ± 19 543.52	40 000 (25 000–56 000)	37 154.88 ± 21 387.96	32 000 (20 000–50 000)

Q1, first quarter; Q3, third quarter.

The validity of UGDS-GS was evaluated via CFA. During construct validity analysis of the 18-item scale, items 1, 3 and 4 were excluded due to a lack of clarity. Subsequently, CFA was conducted with the remaining 15 items that were fitted to the model, with CFI 0.993, TLI 0.991 and RMSEA 0.071. Table 2 shows the item factor loadings (items 1, 3 and 4 were excluded) of UGDS-GS. For known group validity, UGDS-GS scores were compared between different groups to assess the scaleʼs sensitivity to expected variations. There was no statistically significant difference between clinical- (median 25) and community-based adolescents (median 23, *U* = 6204, *P* = 0.08). By contrast, a significant difference was observed between females (median 26) and males (median 21, *U* = 4071.5, *P* < 0.001). Furthermore, the analysis revealed a statistically significant difference between participants under 18 years of age (median 27) and those aged 18 and older (median 23, *U* = 5429.5, *P* = 0.006), indicating that subgroup comparisons based on age were conducted and supported the known-group validity of the scale.

**Table 2:** Item factor loadings of the Utrecht Gender Dysphoria Scale–Gender Spectrum

Items	Factor loading (s.e.)	*P*-value
(2) Every time someone treats me like my assigned sex I feel hurt.	0.820 (0.030)	**<0.001**
(5) A life in my affirmed gender is more attractive for me than a life in my assigned sex.	0.662 (0.043)	**<0.001**
(6) I feel unhappy when I have to behave like my assigned sex.	0.912 (0.017)	**<0.001**
(7) It is uncomfortable to be sexual in my assigned sex.	0.949 (0.011)	**<0.001**
(8) Puberty felt like a betrayal.	0.739 (0.038)	**<0.001**
(9) Physical sexual development was stressful.	0.485 (0.052)	**<0.001**
(10) I wish I had been born as my affirmed gender.	0.775 (0.035)	**<0.001**
(11) The bodily functions of my assigned sex are distressing for me (i.e. erection, menstruation).	0.738 (0.037)	**<0.001**
(12) My life would be meaningless if I would have to live as my assigned sex.	0.963 (0.009)	**<0.001**
(13) I feel hopeless if I have to stay in my assigned sex.	0.975 (0.008)	**<0.001**
(14) I feel unhappy when someone misgenders me.	0.436 (0.062)	**<0.001**
(15) I feel unhappy because I have the physical characteristics of my assigned sex.	0.904 (0.018)	**<0.001**
(16) I hate my birth-assigned sex.	0.944 (0.013)	**<0.001**
(17) I feel uncomfortable behaving like my assigned sex.	0.937 (0.013)	**<0.001**
(18) It would be better not to live than to live as my assigned sex.	0.947 (0.018)	**<0.001**

Bold type indicates statistically significant *P*-values (<0.05).

The criterion validity of UGDS-GS was evaluated using ROC curve analysis, with UGDS serving as the gold standard. The area under the curve (AUC) was 0.947, with a standard error of 0.022 and a highly significant *P*-value (*P* < 0.001), indicating excellent discriminatory ability. The 95% CI for AUC ranged from 0.904 to 0.991, further supporting the robustness of UGDS-GS validity. Because all participants completed both UGDS-GS and the previously validated Turkish version of UGDS – for which a well-established cut-off score was available – ROC analysis was performed using the UGDS classification as the reference. An optimal cut-off value of 42.5 was identified for UGDS-GS, where sensitivity was 80% and specificity 95.9%. The corresponding Youden Index was 0.759, highlighting a strong balance between sensitivity and specificity. This threshold was selected based on the point that maximised the Youden Index, ensuring the best possible trade-off between false positives and false negatives in alignment with the Turkish UGDS classification. These findings demonstrate that UGDS-GS is highly effective in distinguishing between different outcomes when benchmarked against UGDS, thereby confirming its strong criterion validity.

The scores from UGDS-GS exhibited statistically significant yet weak correlations with several RSES subscales, including depressive affect (*r* = 0.196, *P* = 0.002), daydreaming (*r* = 0.185, *P* = 0.005), psychosomatic symptoms (*r* = 0.260, *P* < 0.001), perception of threat in interpersonal relationships (*r* = 0.138, *P* = 0.033), parental involvement (*r* = 0.158, *P* = 0.015) and feelings of psychic isolation (*r* = 0.144, *P* = 0.026). Additionally, UGDS-GS scores showed low to moderate correlations with all subscale scores of YSR, including anxious/depressed (*r* = 0.276, *P* < 0.001), withdrawn/depressed (*r* = 0.260, *P* < 0.001), somatic complaints (*r* = 0.190, *P* = 0.003), social problems (*r* = 0.415, *P* < 0.001), thought problems (*r* = 0.310, *P* < 0.001), attention problems (*r* = 0.334, *P* < 0.001), rule-breaking behaviour (*r* = 0.255, *P* < 0.001), aggressive behaviour (*r* = 0.289, *P* < 0.001), internalising problems (*r* = 0.273, *P* < 0.001), externalising problems (*r* = 0.295, *P* < 0.001), total problems (*r* = 0.359, *P* < 0.001), affective problems (*r* = 0.305, *P* < 0.001), anxiety problems (*r* = 0.249, *P* < 0.001), somatic problems (*r* = 0.156, *P* = 0.016), attention-deficit/hyperactivity problems (*r* = 0.292, *P* < 0.001), oppositional defiant problems (*r* = 0.252, *P* < 0.001), conduct problems (*r* = 0.258, *P* < 0.001), obsessive–compulsive problems (*r* = 0.265, *P* < 0.001) and post-traumatic stress problems (*r* = 0.308, *P* < 0.001). However, no significant correlation was found with the positive qualities subscale (*r* = −0.076, *P* = 0.239). Although these correlations were in the expected direction, their strength was modest. This suggests that, while higher gender dysphoria is related to lower self-esteem and increased psychological difficulties, these associations are not particularly strong, indicating the probable influence of additional moderating or mediating variables beyond gender-related distress alone. According to the cut-off score of UGDS-GS, 10.4% of the sample (*n* = 25) was screened positive for gender dysphoria. This rate was found to be 17.5% (*n* = 21) among clinically based adolescents compared with 3.3% (*n* = 4) among community-based adolescents. When adolescents screening positive for gender dysphoria were compared with the remainder of the sample on the RSES and YSR subscales, significant differences emerged, as illustrated in Table 3.

**Table 3:** Comparison of clinical features of adolescents screened positive for gender dysphoria (GD) based on the Utrecht Gender Dysphoria Scale–Gender Spectrum with others

Variable	Negative screening for GD (*n* = 215)		Positive screening for GD (*n* = 25)		*P*-value
	Mean ± s.d.	Median (Q1–Q3)	Mean ± s.d.	Median (Q1–Q3)	
RSES self-esteem	1.61 ± 1.12	1.3 (0.75–2.33)	2.62 ± 1.30	2.58 (1.58–3.76)	**<0.001**
RSES stability of self-concept	3.81 ± 1.13	4 (3–5)	3.52 ± 1.36	4 (3–4)	0.317
RSES trust in others	1.64 ± 0.81	2 (1–2)	1.92 ± 0.81	2 (1–2)	0.066
RSES sensitivity to criticism	1.78 ± 1.12	2 (1–3)	1.76 ± 1.20	2 (1–3)	0.969
RSES depressive affect	2.43 ± 1.47	2 (1–3)	3.24 ± 1.45	3 (2–4)	**0.008**
RSES daydreaming	1.61 ± 1.44	1 (0–3)	2.64 ± 1.22	3 (2–4)	**0.002**
RSES psychosomatic symptoms	4.26 ± 2.80	4 (2–7)	5.72 ± 3.01	5 (4–8)	**0.020**
RSES perception of threat in interpersonal relationships	1.33 ± 1.02	1.5 (0–2)	1.64 ± 1.15	2 (0–2)	0.140
RSES degree of engagement in discussions	0.68 ± 0.77	0 (0–1)	1.00 ± 0.91	1 (0–2)	0.085
RSES parental involvement	1.70 ± 1.52	1 (1–3)	2.76 ± 1.79	2 (1–4)	**0.003**
RSES relationship with father	1.01 ± 1.21	1 (0–1)	1.09 ± 1.53	1 (0–2)	0.860
RSES feelings of psychic isolation	1.14 ± 0.83	1 (0–2)	1.48 ± 0.71	2 (1–2)	**0.048**
YSR anxious/depressed	62.76 ± 11.29	62 (54–67)	69.08 ± 11.13	66 (60–76)	**0.004**
YSR withdrawn/depressed	64.34 ± 11.86	61 (55–73)	71.32 ± 13.43	66 (61–82)	**0.010**
YSR somatic complaints	59.73 ± 10.27	55 (51–66)	65.04 ± 12.45	65 (52–69)	**0.015**
YSR social problems	59.13 ± 8.72	57.5 (51–64)	67.64 ± 7.47	66 (64–73)	**<0.001**
YSR thought problems	59.51 ± 8.22	58 (53–64)	72.76 ± 12.69	70 (63–84)	**<0.001**
YSR attention problems	60.83 ± 10.53	58.5 (52–66)	68.28 ± 12.31	66 (57–78)	**0.002**
YSR rule-breaking behaviour	54.70 ± 6.00	52 (50–58)	59.64 ± 8.53	59 (53–66)	**0.002**
YSR aggressive behaviour	57.00 ± 7.81	55 (51–60)	64.64 ± 8.90	66 (59–70)	**<0.001**
YSR internalising problems	62.41 ± 11.70	62 (54–69)	70.16 ± 10.01	70 (63–76)	**0.002**
YSR externalising problems	53.57 ± 9.82	54 (46–60)	61.84 ± 11.02	63 (56–68)	**<0.001**
YSR total problems	58.57 ± 10.85	58 (51–65)	69.4 ± 10.14	69 (65–77)	**<0.001**
YSR affective problems	63.55 ± 10.83	58 (51–65)	73.16 ± 12.00	70 (65–82)	**<0.001**
YSR anxiety problems	56.81 ± 7.54	55 (51–59)	60.04 ± 6.53	59 (55–68)	**0.008**
YSR somatic problems	58.74 ± 10.33	54.5 (50–65)	62.84 ± 12.42	60 (53–69)	0.083
YSR attention-deficit hyperactivity problems	56.65 ± 6.76	54 (51–60)	61.88 ± 7.55	63 (57–66)	**0.001**
YSR oppositional defiant problems	57.04 ± 7.63	55 (51–61)	63.16 ± 8.81	65 (55–69)	**0.001**
YSR conduct problems	54.19 ± 6.67	51 (50–56)	61.56 ± 10.44	62 (51–67)	**<0.001**
YSR obsessive–compulsive problems	64.43 ± 36.37	63 (55–68)	67.64 ± 10.05	68 (60–73)	**0.007**
YSR post-traumatic stress problems	63.17 ± 10.25	61 (56–68)	70.2 ± 9.53	70 (63–79)	**0.001**
YSR positive qualities	49.58 ± 9.67	50 (44–56)	45.56 ± 11.58	44 (36–55)	0.065

RSES, Rosenberg Self-Esteem Scale; YSR, Youth Self-Report.

Bold type indicates statistically significant *P*-values (<0.05).

Cronbachʼs alpha value for UGDS-GS was 0.935, indicating high internal consistency. To assess the test–retest reliability of UGDS-GS, the ICC was determined to be 0.884 (95% CI: 0.767–0.944, *P* < 0.001), demonstrating a strong positive agreement between test scores at two different time points. These results suggest that UGDS-GS exhibits consistency and stability over time.

## Discussion

This study evaluated the psychometric properties of UGDS-GS in a sample including both clinical- and community-based groups. The findings demonstrated strong content validity, because a significant majority of experts deemed the items relevant to the construct of gender dysphoria. Construct validity was confirmed through factor analysis, indicating that the scale effectively measured the intended dimension. Notably, UGDS-GS successfully differentiated scores based on demographic factors such as gender and age, highlighting its sensitivity to variations within the population. The scale also exhibited excellent criterion validity, suggesting that it is highly effective in identifying individuals with gender dysphoria. Furthermore, UGDS-GS demonstrated high internal consistency and strong test–retest reliability, indicating stability in the results over time and across different assessments. These results suggest that UGDS-GS is a valuable and reliable tool for evaluation of gender dysphoria in Turkish adolescents, although further research may be beneficial to enhance its applicability across varying contexts and populations.

The content validity of the Turkish adaptation of the scale was evaluated, with 69% of experts rating the items as essential. Although this value exceeds the minimum threshold of 0.67 for acceptability,^22^ it remains below the commonly recommended standard of 0.75. Notably, expert feedback indicated that items related to the gender affirmation subdimension – particularly those using terms such as ‘affirmed genderʼ – were occasionally viewed as unclear or unfamiliar. This probably reflects a cultural and linguistic gap in the understanding and usage of gender-neutral terminology in Turkish clinical settings. While these items are conceptually valid in the original context, further cultural adaptation may be required to enhance their clarity and relevance for local professionals. These findings raise questions about the construct validity of the gender affirmation dimension within this sample. Unlike the original study, which included participants across the gender spectrum (cisgender, transgender, non-binary and lesbian, gay, bisexual and questioning), the present sample consisted of adolescents from a clinical setting and community-based university students, many of whom may be cisgender.^15^ As such, terms like ‘affirmed genderʼ may not resonate meaningfully with all participants. Indeed, in the original study, one cisgender respondent also noted confusion regarding this term, underscoring its conceptual complexity even in more gender-diverse populations.^15^ This sampling difference may also explain why items related to gender affirmation were statistically weaker in the Turkish context. Participants who do not actively experience gender incongruence may find such items less personally relevant or harder to interpret, contributing to their lower factor loadings. Future validation studies including larger samples of gender-diverse adolescents in Turkey could help reassess the relevance and retention of these items. Given that UGDS-GS was designed as a two-subscale measure – dysphoria and affirmation – our findings highlight the importance of further evaluation of whether both dimensions are equally interpretable and valid in culturally specific contexts.^14,15^

Ultimately, the findings of our study support the unidimensional structure of the 15-item Turkish UGDS-GS, focusing on gender dysphoria rather than gender affirmation. Gender affirmation refers to the process of being recognised and supported in oneʼs gender, encompassing social, legal, medical and psychological dimensions.^23^ It plays a critical therapeutic role for many individuals with gender dysphoria. However, because gender affirmation often represents a later stage in the gender identity journey – especially during adolescence – and considering that many participants in our sample were probably cisgender, items addressing affirmation may not have been fully understood or endorsed.

Additionally, research shows significant differences in the utilisation of transgender health services among cisgender, transgender and non-binary individuals, particularly with respect to body satisfaction and perceived gender congruence.^24^ For instance, binary transgender individuals are more likely to view transition-related medical interventions (e.g. hormone therapy, surgery) as essential.^25^ This variation underscores the individualised nature of gender affirmation and highlights why affirmation-related items may have functioned differently in our sample.

The removal of items 1, 3 and 4 – originally designed to capture aspects of gender affirmation – thus alters the factor structure of UGDS-GS. Although the revised 15-item version demonstrates strong psychometric properties, this structural shift may have compromised the scaleʼs alignment with the original theoretical framework, which emphasised both dysphoria and affirmation experiences. The diminished representation of affirmation could have limited the scaleʼs capacity to capture the full spectrum of gender-related experiences, especially for non-binary and transgender youth. Future research should critically evaluate whether the unidimensional version retains the conceptual integrity and inclusivity of the original scale, and further explore how affirmation is understood and experienced across different gender identities in Turkish adolescent populations.

The data regarding the validity and reliability of the Turkish UGDS-GS were consistent with those from the original study and its adaptations in other languages, with the exception of items related to gender affirmation.^14,26,27^ In our study, 10.4% of the overall sample was screened positive for gender dysphoria according to the cut-off score of the Turkish UGDS-GS, with a higher rate of 17.5% among clinically based adolescents compared with 3.3% among community-based adolescents. It is important to emphasise that this figure reflects results from a screening tool and does not indicate epidemiological prevalence. The sample was not representative of the general adolescent population in Turkey, and the rates reported should be interpreted as indicative of potential gender dysphoria within this specific research context, rather than as diagnostic or population-based prevalence. The prevalence of gender dysphoria varies widely, influenced by the methodologies of studies and the cultural and geographical differences in social acceptance and awareness.^28^ In Taiwan, a retrospective medical record review indicated that the number of gender dysphoria cases approximately doubled between 2010 and 2019, with a prevalence in 2019 of 7.4 per 100 000 for assigned males and 3.2 per 100 000 for assigned females.^29^ Conversely, a survey conducted in the USA analysed 3168 responses and found incongruence between gender identity and gender assigned at birth in 291 participants, representing a prevalence of 9.2%.^30^ The high prevalence rates observed in our sample may be attributed not only to increased public awareness, greater media representation, reduced stigma and a better understanding of gender and gender diversity, but also to the use of gender-neutral language in UGDS-GS. This transition to inclusive language can promote a more accurate representation of gender identities and experiences, encouraging individuals to engage with the assessment more openly.

The primary aim of the assessment using UGDS-GS was to identify gender dysphoria in youth and to provide support for the associated physical and mental health concerns, all within an open-minded and compassionate environment.^31^ The prevalence of mental disorders, self-harm and suicidality is known to be increased among adolescents experiencing gender dysphoria.^32,33^ While this association is well documented, the present cross-sectional design does not allow for causal inference regarding the direction or mechanisms underlying this relationship. Participants who screened positive for gender dysphoria demonstrated a higher prevalence of emotional and behavioural problems, including anxiety, depression, social withdrawal and interpersonal difficulties. These findings suggest a potential link, although it remains unclear whether psychological distress precedes or results from gender dysphoria. Contributing factors may include minority stress, shame and the psychological burden of secrecy and social stigma.^28,34^ In addition, these individuals showed elevated levels of attention deficits, thought problems, rule breaking and aggressive behaviour. Symptoms associated with disorders such as oppositional defiant disorder, conduct disorder, obsessive–compulsive disorder and post-traumatic stress disorder were also more pronounced.

The weak-to-moderate correlations observed between UGDS-GS and the RSES and YSR subscales are consistent with the multifactorial nature of psychological constructs such as self-esteem, depression and anxiety. Although gender dysphoria may play a role in psychological distress, it is one of many interacting influences. Therefore, the modest strength of these associations does not undermine the validity of the scale but rather reflects the complex interplay of individual, social and environmental factors affecting mental health. The lack of direct gender identity data in the current study may have further increased sample heterogeneity, potentially weakening observable relationships between gender dysphoria and psychosocial outcomes. Interestingly, symptoms of attention-deficit/hyperactivity disorder were also higher in the gender dysphoria-positive group, consistent with existing research.^35^ These comorbidities may be driven by shared underlying mechanisms. Additionally, these adolescents reported significantly lower self-esteem, increased daydreaming and more severe psychosomatic symptoms. Reduced parental involvement and heightened feelings of psychic isolation further underscore the psychological vulnerability of this population. However, critical psychosocial variables – such as religiosity, family support and experiences of stigma – were not assessed in this study. Future research including these variables, along with longitudinal designs, is essential to clarify the directionality and underlying mechanisms of these associations. A psychosocially supportive, non-pathologising and family-inclusive healthcare model – as exemplified by UGDS-GS – remains crucial for improving outcomes in gender-diverse youth.^31^

Despite the valuable insights gained from this study, several limitations must be acknowledged. First, participants were asked about their biological sex but not their gender identity, sexual orientation or religious beliefs. This omission reflects concerns about stigma in cultural contexts where gender diversity and sexuality are often considered taboo. However, it represents a critical limitation, particularly given that the aim of the study was to validate a tool designed to assess gender dysphoria. Without direct data on gender identity, it is not possible to fully evaluate how the scale performs across diverse identities, especially among non-binary and transgender individuals. This limits interpretation of the scaleʼs sensitivity and specificity beyond the cisgender population. Second, the use of self-report measures introduces the potential for bias, because participants may under- or over-report symptoms due to social desirability or stigma-related concerns. Third, although the sample included both clinical- and community-based participants, all were from urban areas; this may have limited the generalisability of findings to rural or more culturally conservative regions. Additionally, as gender identity was not directly assessed, the sample may be skewed toward cisgender individuals, which could have influenced both the observed prevalence and the scaleʼs ability to reflect the experiences of gender-diverse youth. Finally, the cross-sectional nature of the study precludes any inference of causality regarding the relationship between gender dysphoria and emotional or behavioural problems. Future research should adopt more diverse and representative samples and consider longitudinal designs to better capture the evolving and multifaceted nature of gender dysphoria and its psychological correlates.

In conclusion, this study underscores the strong psychometric performance and clinical applicability of the Turkish adaptation of UGDS-GS in assessing gender dysphoria among adolescents. The scale demonstrated high reliability and validity – specifically in terms of content, construct and criterion metrics – making it a robust tool for both clinical screening and research purposes. Importantly, UGDS-GS offers an inclusive, gender-neutral framework that addresses a critical gap in assessment tools available in the Turkish context. The study also sheds light on the complex psychological profiles of adolescents who screened positive for gender dysphoria, including elevated emotional and behavioural difficulties that may be influenced by minority stress, stigma and internalised conflict. These findings support the importance of early identification and intervention for youth experiencing gender-related distress. However, given the cross-sectional nature of the study, no causal inferences can be made regarding the relationship between gender dysphoria and associated mental health challenges. Nevertheless, the insights gained lay the groundwork for more nuanced, longitudinal investigations that can further illuminate these dynamics over time. By providing a culturally and linguistically validated tool, this study contributes meaningfully to the advancement of gender-affirming care in Turkey. Continued research – particularly using longitudinal and mixed-methods approaches – will be essential to deepen our understanding, assess intervention outcomes and inform best practices. Ultimately, fostering inclusive and stigma-free healthcare environments remains vital to supporting the well-being of all gender-diverse youth navigating their identities.

## Data Availability

The data supporting this studyʼs findings are available on request from the corresponding author. The data are not publicly available due to preserving the privacy of research participants.
